# Influence of moderately warm and extremely cold climate on properties of basalt plastic armature

**DOI:** 10.1016/j.heliyon.2018.e01060

**Published:** 2018-12-21

**Authors:** V.O. Startsev, M.P. Lebedev, A.K. Kychkin

**Affiliations:** a All-Russian Scientific Research Institute of Aviation Materials, 17, Radio Street Moscow, Russia; b V.P. Larionov Institute of Physical-Technical Problems of the North, Siberian Branch of the Russian Academy of Science, 1, Oktyabrskaya Street, Yakutsk, Russia

**Keywords:** Materials science

## Abstract

Rods of basalt plastic armature (BPA) of a periodic profile with diameters of 6, 8, 10, 16 and 20 mm on the basis of an epoxy matrix were exposed for 30 months on open stands in a moderately warm marine climate of Gelendzhik and 28 months on similar stands in an extremely cold climate of Yakutsk. For initial and exposed BPA samples, changes of mechanical parameters were determined. After the exposure in Yakutsk, an increase of ultimate compression strength by 4–12% is found, depending on the diameter of rods. After the exposure in Gelendzhik, this indicator decreases by 10–17%. Using methods of thermomechanical analysis and dynamic mechanical analysis, linear thermal expansion coefficient and glass transition temperature of epoxy matrix were measured. In an initial state, a transition from a glassy state to a highly elastic one is discovered: an ${\text{α}}_{1}$-transition at 118 °C and an ${\text{α}}_{2}$-transition at 149 °C. After climatic action, there were detected a shift of ${\text{α}}_{1}$-transition to low temperatures and a shift of ${\text{α}}_{2}$-transition to higher temperatures. Reasons for the change of temperatures of transitions are weakening of a molecular layer and post-curing of the epoxy matrix in a surface layer. These effects are accompanied by an increase of the linear thermal expansion coefficient, moisture diffusion coefficient and maximum moisture saturation after the climatic action on BPA. Fractographic studies discovered presence of pores in a structure of BPA with sizes up to 10–20 μm, a quantity and size of which increase by 20–40% after the climatic action. In general, the studied BPA has high climatic stability and can be used for a long time under the extreme climatic conditions.

## 1 Introduction

The basalt plastic armature of periodic profile (BPA) is used in construction of industrial structures, electric power facilities, in road construction, in light arched building structures, to which demands for climatic and corrosion resistance are made. Advantages of polymeric materials reinforced with basalt fibers are discussed in a number of works [1, 2, 3, 4, 5, 6, 7, 8, 9, 10] according to mechanical parameters, cost, resistance to aggressive environments, and biological resistance.

“TBM” LLC (Limited Liability Company) (Yakutsk) produces basalt plastic rods of periodic screw profile, which according to mechanical strength or parameters of adhesion with concrete surpass characteristics of rods made from reinforcing steel (Technical Specifications 2296-001-86166796-2013 “Nonmetallic composite armature made from basalt plastic”).

Basalt plastic rods are especially useful as reinforcing elements of concrete structures. In [2] it is shown that the basalt plastics are resistant to action of water and chemically active solutions. After 250 days of being in these media at room temperature, ultimate tensile strength and modulus of elasticity change by 10–20%. According to data [3], plates and pressed layers of impregnated basalt fiber are resistant to the action of dry and wet climate, however their mechanical parameters are unstable and fluctuate within 30–40%. In paper [4] durability and destruction of basalt plastic are compared to similar parameters of glass-reinforced plastics and carbon plastics after being in a humid environment and chemically active solutions. It is shown that systems reinforced by basalt fibers have high resistance to these aggressive influences. A similar comparison was made in [5, 6, 7] and it is shown that the ultimate tensile strength, ultimate bending strength, ultimate shear strength, and moduli of elasticity in tension, bending and shear are not inferior in their durability to the glass-reinforced plastics. In [8, 9, 10], resistance of basalt plastics to the action of low, high temperatures and thermal cycles was studied and stability of these materials to the indicated effects was shown. In [11, 12], analogous conclusions about the high stability of BPA against the action of aggressive media were obtained after evaluating the effect of water, salt and alkaline solutions at normal and increased temperatures on the mechanical properties of rods with diameters of 11–12 mm. Samples of basalt plastics based on a polyester matrix sorbed up to 2% of normal and sea water at room temperature according to [13]. The tensile strength, bending strength and impact strength were reduced by 50–60%, especially for samples, in which the basalt fibers were treated with solutions of H_2_SO_4_ and NaOH before production of composites.

Similar mechanical properties of basalt plastics reinforced with finely chopped roving, fabrics or uniaxially oriented continuous fibers, are compared with carbon plastics and the glass-reinforced plastics based on unsaturated polyesters, epoxyphenolic and phenol-formaldehyde matrices [14]. It is shown that during 12 months of climatic tests under conditions of the South Caucasus there was observed a decrease of strength, depending on a type of polymer matrix and magnitude of applied tensile stress. It is noted that aging processes develop on surfaces of the samples, but no experimental proof of this statement is given.

To improve the mechanical properties and stability of BPA, heat-resistant polymer matrices [15, 16] with modifying finely dispersed additives are used. The properties and structure of the polymer matrix in boundary layers with the basalt fiber are studied by methods of electron microscopy, dynamic mechanical analysis, differential scanning calorimetry, thermogravimetry and other sensitive methods [15, 16].

An urgent problem of use of BPA in construction is confirmation and justification of retention of properties at a high level during long-term operation in a variety of climatic conditions. However, in all the works listed [1, 2, 3, 4, 5, 6, 7, 8, 9, 10, 11, 12, 13, 14, 15, 16], there is no information about a change of properties of BPA for construction purposes under the action of natural climatic conditions of different zones. In [17], the strength of basalt, carbon and glass fibers was studied in accelerated tests simulating the natural weather conditions, but conclusions obtained were not verified by direct climatic tests.

In this regard, an experimental evaluation of the climate resistance of BPA was carried out after the exposure in the extremely cold (Yakutsk) climate and moderately warm marine one (Gelendzhik). The main task of the presented work is to identify the effects of climatic aging in BPA at early stages of exposure using sensitive physical methods.

## 2 Experimental

### 2.1 Materials

For climate testing, BPA was prepared, being unidirectional basalt plastic rods of the periodic profile with the diameters of 6, 8, 10, 16, and 20 mm “TBM” LLC produced the armature on a technical line “Struna” in accordance with Technical Specifications 2296-001-86166796-2013 “Non-metallic composite armature made from basalt plastic”.

The following components were used to produce BPA.

A starting binder, which is based on epoxidation resin ED-22, cured by iso-methyltetrahydrophthalic anhydride (iso-MTHPA) in presence of an accelerator 2,4,6-tris (dimethylaminomethyl)phenol (UP-606/2) was prepared using a formulation according to RTP-SP2- 20994511-1999T (Table 1).

**Table 1:** Composition of formulation of epoxy anhydride binder EDI.

Names of components	Quantity, mass%
Epoxy-diane resin ED-22	100
Hardener iso-MTHPA	75
Accelerator UP-606/2	1.41

Table 2 shows the main characteristics of this epoxy resin.

**Table 2:** Characteristics of epoxy-diane resin ED-22.

Parameter	Value of parameter	
	Requirements of GOST 10587-84	Measured values
Appearance	Transparent, viscous liquid without visible mechanical inclusions and traces of water	Corresponds
A color on an iron-cobalt scale	Not more than 4	1
A mass fraction of epoxy groups,%	19.9–22.0	21.4
A mass fraction of chloride ion,%	Not more than 0.003	0.0009
A mass fraction of saponifiable chlorine,%	Not more than 0.5	0.4
A mass fraction of volatile substances,%	Not more than 0.5	0.3
Dynamic viscosity at 25°С, Pa·s	12–18	16
Mass fraction of hydroxyl groups,%	Not more than 1.7	1.5

Epoxy resins are reactive oligomers that transform into an infusible and insoluble state only under the action of hardeners.

Iso-MTHPA is used as the hardener of “hot” curing of epoxy resins and compositions based on them. It ensures high physical and mechanical characteristics of cured systems, excellent waterproof properties, good electrical characteristics, and the climatic stability.

The main characteristics of iso-MTHPA are shown in Table 3.

**Table 3:** Characteristics of hardener iso-MTHPA.

Parameter	Value of parameter	
	Requirements of technical specifications 6-09-124-91	Measured values
Appearance	Transparent liquid without mechanical inclusions from light yellow to light brown colors	Corresponds
A mass fraction of the main substance,%	Not less than 98.5	99.2
Viscosity according to a viscometer V3-4 at 20 °C, s	Not more than 30.0	28
Gelatinization time at 150 °C, h	6–12	8
A mass fraction of acid,%	Not more than 3.5	2

Since the curing of epoxy resins by anhydrides proceeds extremely slowly, accelerators are usually introduced into these systems. A choice of 2,4,6-*tris*(dimethylaminomethyl)phenol (UP 606/2) as an accelerator is due to its high catalytic activity associated with presence in this compound of three tertiary amino groups and an acidic phenolic hydroxyl at the same time:

**Figure:**
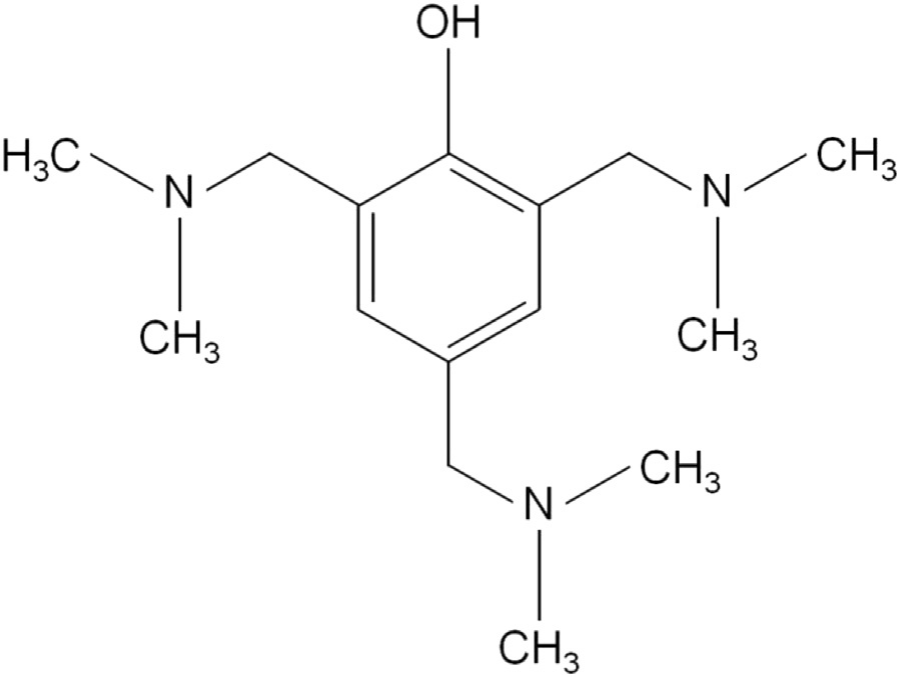

However, it manifests itself at rather high temperatures (above 100 °C). At processing temperatures, processability of the binder in the presence of the accelerator is maintained. Characteristics of UP-606/2 curing accelerator are presented in Table 4.

**Table 4:** Characteristics of UP-606/2 curing accelerator.

Parameter	Value of parameter	
	Requirements of technical specifications 2495-449-05742686-2003	Measured values
Appearance	Viscous liquid from yellow to brown colors	Corresponds
A mass fraction of the main substance,%	Not less than 93	98.4
A mass fraction of titrated nitrogen,%,	Not less than 8	14
Density at a temperature of 25 °C, kg/m^3^	6–12	10

A fragment of chemical knots of a spatial network in the anhydride curing:

**Figure:**
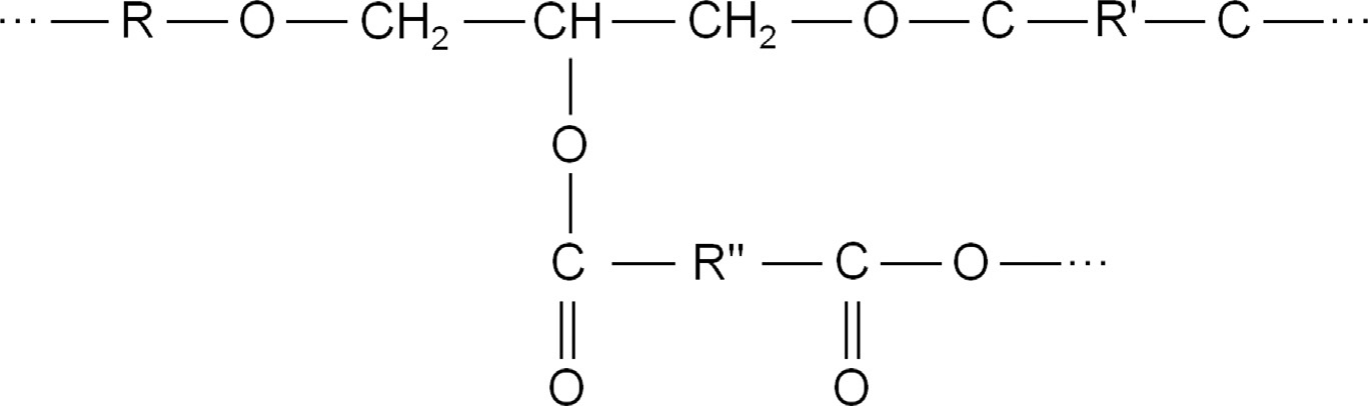

An ester group formed as a result of curing is resistant to the action of organic and many inorganic acids, but is destroyed by alkalis, but thermal stability and electrical insulation properties are higher than with the use of amine hardeners.

**Figure:**
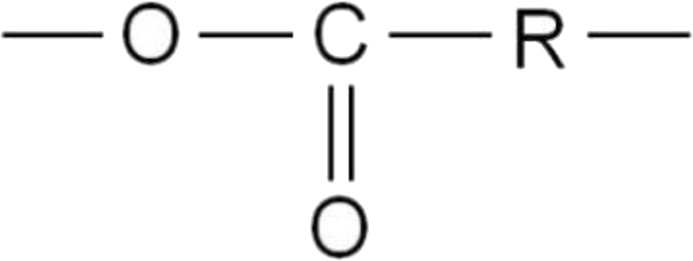

Basalt roving RBN 13-2400-4S of production by “TBM” LLC (Yakutsk). Physical and mechanical parameters of this roving RBN 13-2400-4C are shown in Table 5.

**Table 5:** Physical and chemical properties of basalt roving.

Name of parameter	RBN 13-2400-4S
1. Nominal linear density of roving with an allowable deviation in linear density from nominal, tex	2400 ± 60
2. A diameter of an elementary fiber, μm	13
3. Breaking load, mN/tex, not less than	320
4. A mass fraction of substances removed during calcination,%, not less than	0.3
5. A mass fraction of moisture,%, not more than	1.0

### 2.2 Environmental conditions

**Fig. 1:**
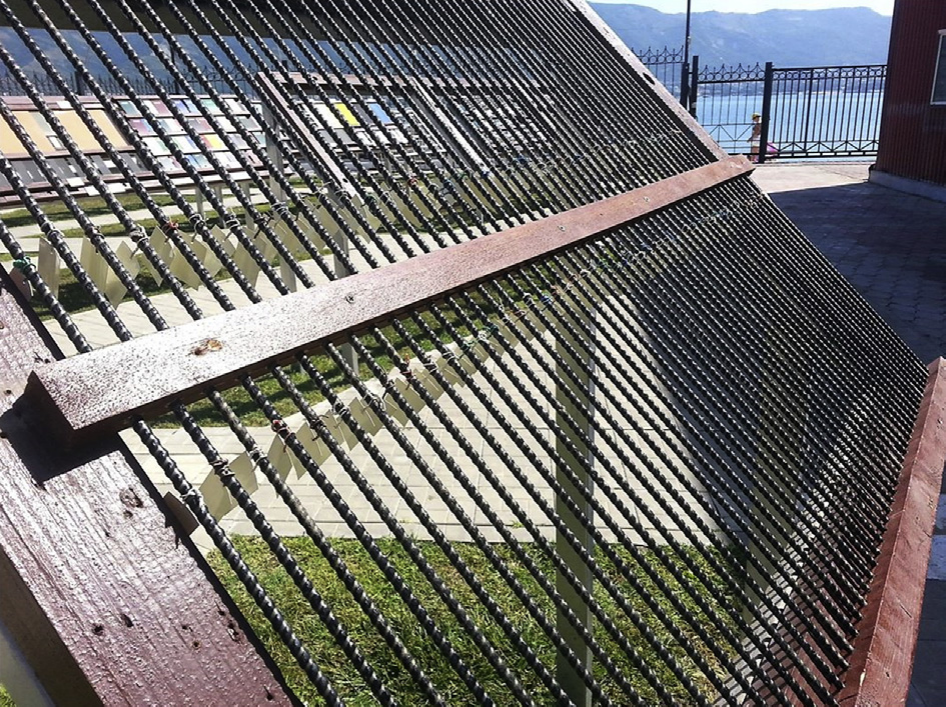
Appearance of atmospheric stand for exposure of BPA in open conditions of moderately warm climate of Gelendzhik.

**Fig. 2:**
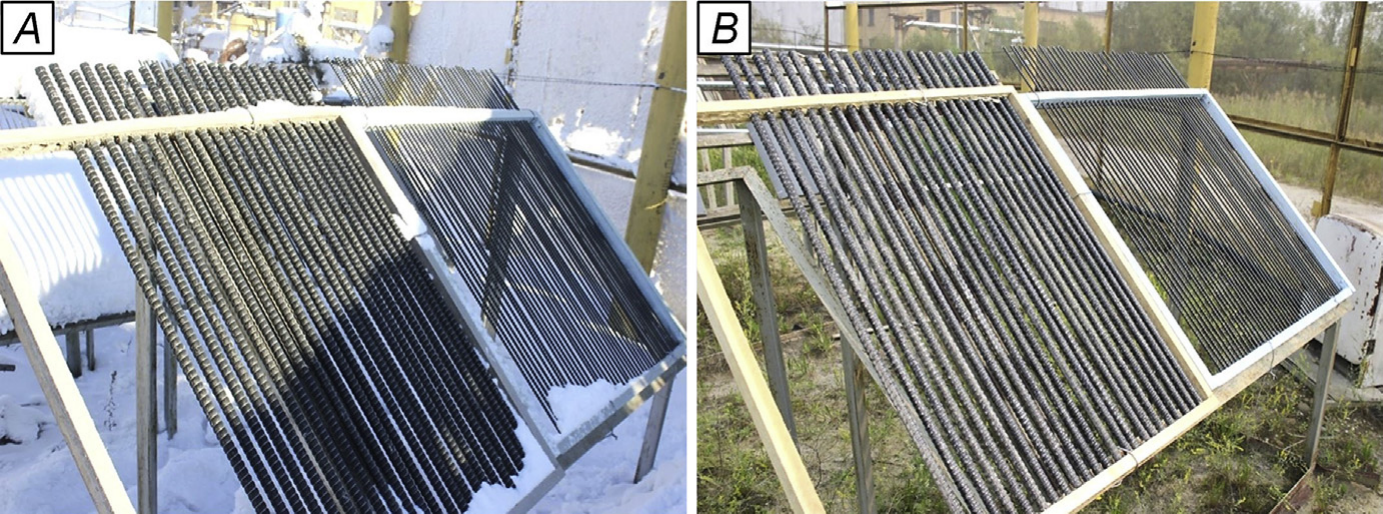
Exposure of BPA in open climatic conditions of Yakutsk in winter (A) and summer (B).

**Fig. 3:**
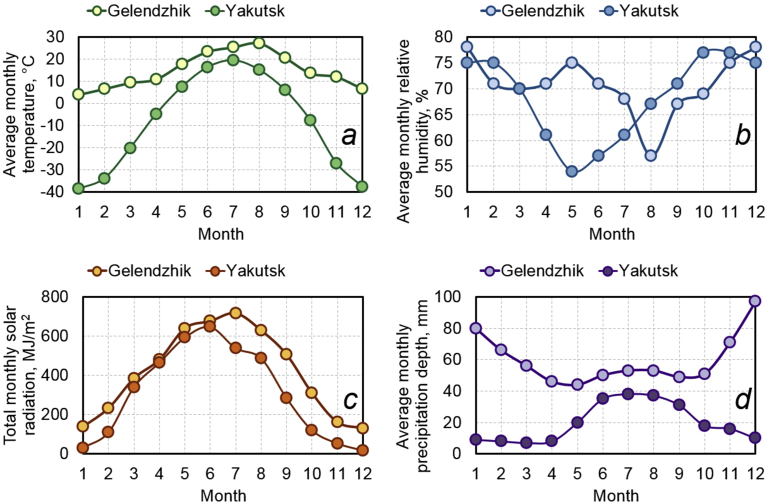
Average monthly climatic parameters of Gelendzhik and Yakutsk: temperature (a), relative humidity (b), total solar radiation (c), precipitation depth (d).

To assess an effect of solar radiation, temperature, humidity, precipitation and other aggressive climate factors, the rods of BPA of the periodic profile with diameters of 6, 8, 10, 16 and 20 mm were exposed during 30 months on the open stands in the moderately warm marine climate of Gelendzhik and 28 months on the similar stands in the extremely cold climate of Yakutsk (Figs. 1 and 2). Average monthly climatic parameters (Fig. 3) characterize well-defined seasonality of Gelendzhik and Yakutsk. An average annual air temperature in Gelendzhik is 14.8 °C, in Yakutsk minus 8.8 °C. The city of Yakutsk is in a zone of a sharply continental climate with very cold winters and a relatively hot and short summer. The minimum recorded temperature is –64.4 °C. An annual amplitude of temperatures between the highest and lowest values exceeds 100 °C. In winter, in Yakutsk at a temperature below –40 °C, all water vapor being in the air crystallizes and forms fogs. An average wind speed is 1.8 m/s.

The average annual relative humidity in Gelendzhik and Yakutsk differs slightly (71% and 68% respectively). During a year, precipitation in Gelendzhik is greater than in Yakutsk by a factor of 3.3 (796 mm and 237 mm, respectively). A doze of annual total solar radiation in Gelendzhik is 5004 MJ/m^2^, which is by 36% higher than in Yakutsk (3680 MJ/m^2^).

### 2.3 Tensile and bending tests

**Fig. 4:**
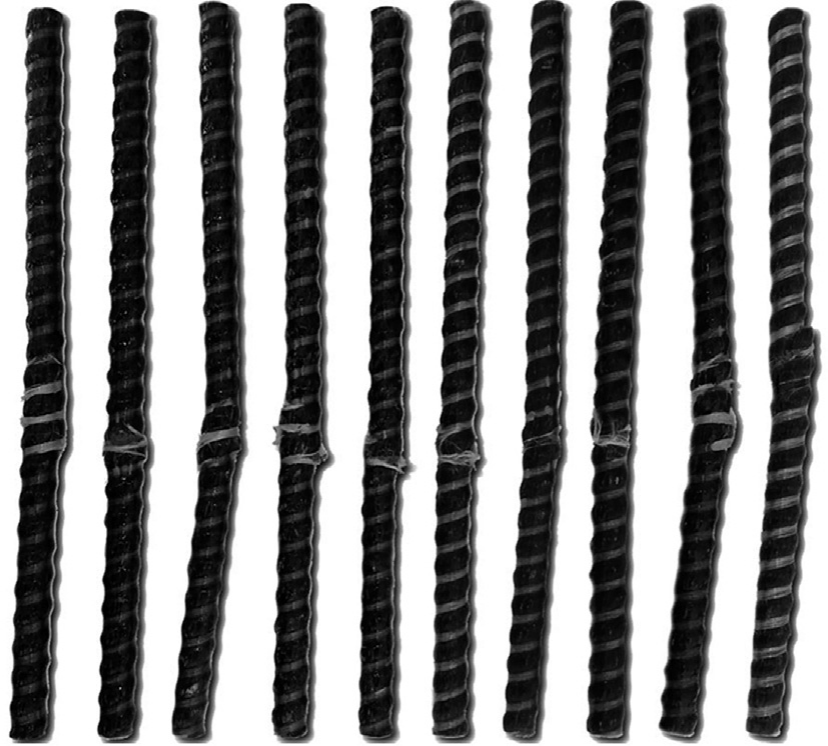
Fracture of series of samples when measuring ultimate compression strength.

To control the mechanical properties of BPA, compression strength $\sigma_{c}$ and buckling strength $\sigma_{b}$ (Russian standard GOST 31938-2012) were determined. Mechanical loading was carried out on a universal test machine *Z100 Zwick/Roell* before the samples were destroyed in a working area. A typical example of fracture of the samples during compression tests is shown in Fig. 4.

### 2.4 Dynamic mechanical analysis

According to [18, 19, 20], the glass transition temperature $T_{g}$ of the polymer matrix is a sensitive indicator of the climatic aging of polymer composite materials (PCM). The method of dynamic mechanical analysis (DMA) was used to determine this parameter. With the help of an analyzer *DMA 242D Netzsch*, a dynamic elastic modulus $E^{\prime }$ and dynamic loss modulus $\;E^{\prime \prime }$ were measured in a temperature range from 25 °C to 200 °C at a rate of 3 °C/min. Samples in a form of strips with sizes of 40 × 4 × 1 mm were cut from a central part of BPA along an rod axis. Measurements were made with a frequency of forced oscillations of 0.5 Hz, and a dynamic load of 5 N at an amplitude of oscillations up to 200 μm. A single-cantilever attachment scheme was used, in which one end of the sample was rigidly fixed in a gripper, and the other end of the sample was clamped in the movable cantilever. A calculation of the parameters $E^{\prime }$ and $E^{\prime \prime }$ was carried out using formulas

(1) $$E^{\prime }=\frac{ANL^{3}}{BH^{3}}\cos\;\delta$$

(2) $$E^{\prime \prime }=\frac{ANL^{3}}{BH^{3}}\sin\;\delta$$

where N is an axial force, L is a length of free part of the sample, B is a width of the sample, H is a thickness of the sample, A is the amplitude of oscillations, δ is an angle of a phase shift between the stress and strain of the sample.

### 2.5 Thermal expansion

Control of thermal expansion of the samples of BPA was carried out during heating over a wide range of temperatures using a thermomechanical analyzer *TMA PTLT 600* of a company *Linseis*. In a module of this analyzer, a measuring indenter was pressed against the sample with a constant load of 0.5 N applied to an end of the sample with the diameter from 6 mm to 20 mm and a height of 10 mm. Movement measurements of the indenter were controlled with an accuracy of 10^−4^ mm. The temperature in a measuring cell was automatically increased at a rate of 3 °C/min between 25 °C and 200 °C. Results were processed using *Linseis Data Evaluation* software for Windows, version 3.00. In the sort of measurement used, the thermomechanical analyzer operated as a highly sensitive linear dilatometer. The movement of the indenter in a direction of action of a small load over a wide range of temperatures from 200 °C to 300 °C was negligibly small, and when the temperature was increased, micromovements caused by the thermal expansion of the sample were recorded.

### 2.6 Sorption and diffusion of water

**Fig. 5:**
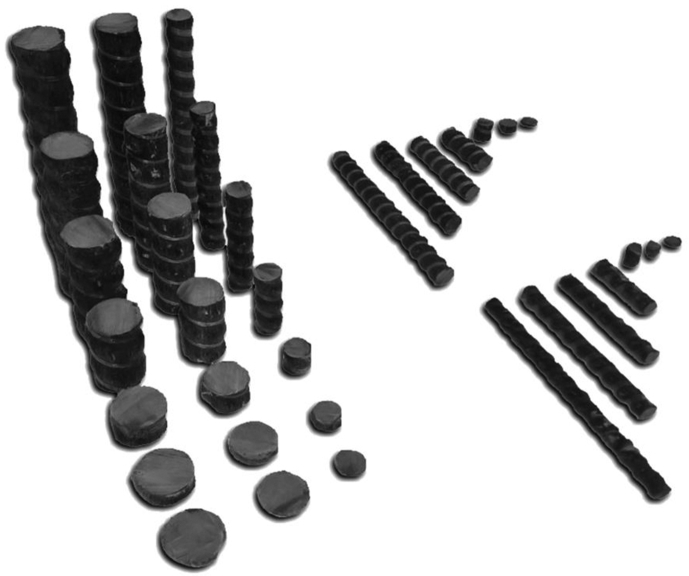
Set of samples of BPA for determination of characteristics of moisture transfer.

It was proved in [21] that the moisture diffusion coefficient D and the maximum moisture content $M_{\infty }$ are parameters sensitive to the climatic aging of PCM. To assess possible physicochemical and structural transformations during the exposure of BPA in the open climatic conditions, kinetics of moisture transfer was studied. From the initial and exposed to the climatic conditions rods of diameters 6, 8, 10, 16, and 20 mm, 3 samples of lengths 2, 5, 10, 30, 50, 70, and 100 mm were cut out (Fig. 5).

The samples were previously dried in an oven at 60 °C over silica gel for 6 days, then they were held at the same temperature and relative humidity of 98 ± 2% in a desiccator above a surface of water. At the same time, measurements of masses of the samples were periodically carried out using analytical scales with an accuracy of 10^−4^ g and their geometric sizes with an accuracy of 0.001 mm. After 63 days of humidification, a process of moisture sorption was stopped and the samples were dried in the oven at the temperature of 60 °C during 35 days.

### 2.7 Fractography

To evaluate the effect of exposure on the structure of BPA, microsections of cross-sections of the rods with the diameters of 8, 10, 16, and 20 mm were studied. Places of saw cuts were thoroughly polished. The fractographic studies of epoxy polymers were carried out with the help of an optical stereomicroscope Olympus SZX-10 with a magnification of 20–50.

## 3 Results and discussion

### 3.1 Mechanical properties

**Fig. 6:**
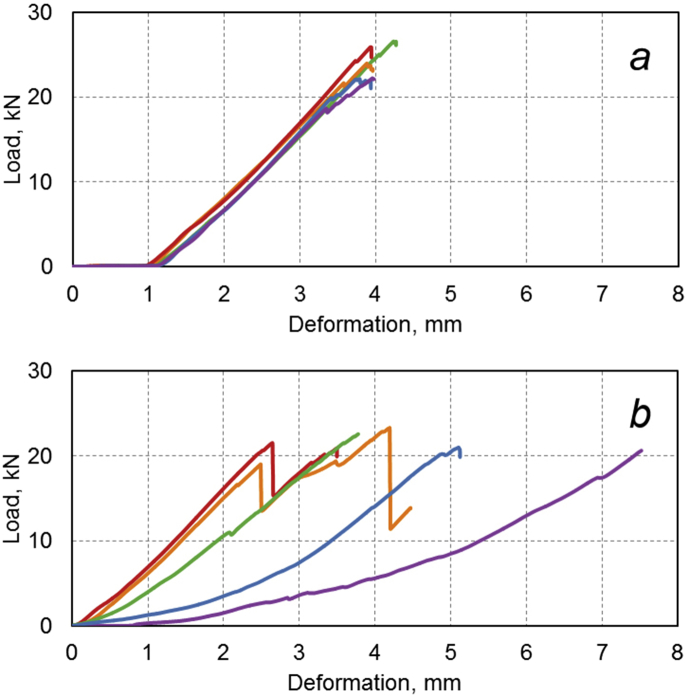
Load diagrams when compressing samples of BPA with diameter of 8 mm in initial state (a) and after 28 months of exposure in Yakutsk (b).

Compression and bending tests are the main and most common methods of control of the mechanical properties of BPA [14, 15, 16, 17]. A typical example of load diagrams of the samples with the diameter of 8 mm is shown in Fig. 6.

Presence of periodic relief on the surface of BPA makes it difficult to determine the compression strength $\sigma_{c}$ and the buckling strength $\sigma_{b}$ due to large spreads of these parameters (Fig. 6b). When analyzing the load diagrams, it is necessary to take into account emissions associated with the destruction of the rods near the ends, causing decreases of the load. A comparison of values of ultimate compression strength of the initial samples and the samples exposed in the cold climate (Table 6) revealed an increase of this parameter by 4–12%, depending on the diameter of the rods. After the exposure in the moderately warm climate, the ultimate compression strength is reduced by 10–17%.

**Table 6:** Mechanical properties of BPA in initial state and after exposure in Gelendzhik and Yakutsk (minimum and maximum (numerator), average (denominator)).

Conditions of tests	Diameter, mm	Buckling strength limit $\sigma_{b}$, MPa	Ultimate compression strength $\sigma_{c}$, MPa
In initial state	6	$\frac{1062-1406}{1209}$	$\frac{354-474}{410}$
	8	$\frac{649-862}{764}$	$\frac{418-519}{466}$
	10	$\frac{298-862}{559}$	$\frac{400-471}{432}$
Full-scale exposure in conditions of moderately warm climate (Gelendzhik) during 30 months	6	$\frac{831-1259}{1094}$	$\frac{368-463}{427}$
	8	$\frac{464-883}{658}$	$\frac{392-452}{420}$
	10	$\frac{358-699}{457}$	$\frac{343-378}{360}$
Full-scale exposure in conditions of extremely cold climate (Yakutsk) during 28 months	6	$\frac{433-1237}{780}$	$\frac{297-509}{428}$
	8	$\frac{843-960}{902}$	$\frac{396-450}{414}$
	10	$\frac{196-1004}{639}$	$\frac{368-509}{452}$

According to the results of buckling tests, it is established that average values of $\sigma_{b}$ of BPA samples with the diameters of 8 mm and 10 mm increase after the exposure in Yakutsk, but decrease after the aging in Gelendzhik for the rods with the diameters of 6, 8, and 10 mm.

A possible reason for the observed changes is the physicochemical transformations in the epoxy matrix in a surface relief layer of the rods due to the action of moisture, solar radiation and surface heating in the summer. However, the large spreads of the measured parameters $\sigma_{c}$ and $\sigma_{b}$ do not allow orientating towards the results of the mechanical tests to justify conclusions about mechanisms of the climatic aging of BPA.

### 3.2 Dynamic mechanical analysis

**Fig. 7:**
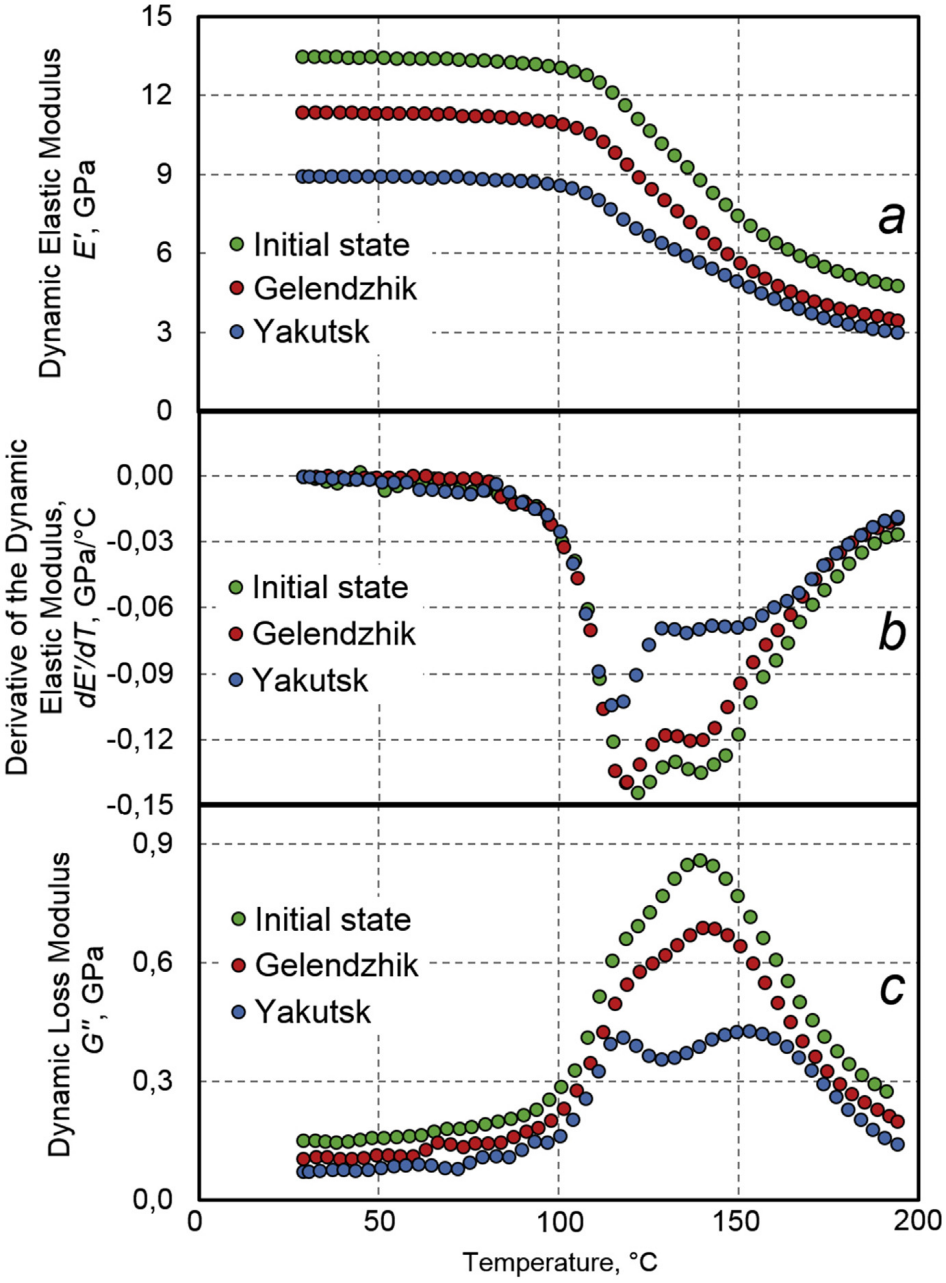
Temperature dependences of dynamic elastic modulus (a), derivative of dynamic elastic modulus (b), and dynamic loss modulus (c) of samples of BPA with diameter of 6 mm in initial state, after 30 months of exposure in moderately warm climate of Gelendzhik and after 28 months of full-scale exposure in extremely cold climate of Yakutsk. DMA measurements are performed at frequency of 0.5 Hz.

Fig. 7 shows temperature dependences of the dynamic elastic modulus $E^{\prime }$, a temperature derivative of the dynamic elastic modulus $dE^{\prime }/dT$, and the dynamic loss modulus $E^{\prime \prime }$ of BPA samples with the diameter of 6 mm in the initial state, after 30 months of full-scale exposure in Gelendzhik and after 28 months of full-scale exposure in Yakutsk. DMA measurements are performed at a frequency of 0.5 Hz. The glass transition temperature $T_{g}$ of the epoxy matrix of BPA is defined as the minimum point on the graph $dE^{'}/dT$ and as the maximum point on the graph $E^{\prime \prime }$ similar to [22]. The glass transition temperature determined by these two criteria is shown in Table 7.

**Table 7:** Effect of exposure in moderately warm and extremely cold climate on temperatures of relaxation α-transitions of epoxy matrix of BPA according results of DMA.

Conditions of tests of BPA	Number of peak	Temperature of relaxation α-transitions, °C					
		0.5 Hz		5 Hz		50 Hz	
		According to $dE^{\prime }/dT$	According to $E^{\prime \prime }$	According to $dE^{\prime }/dT$	According to $E^{\prime \prime }$	According to $dE^{\prime }/dT$	According to $E^{\prime \prime }$
Initial state	$\alpha_{1}$	118	119	122	124	124	129
	$\alpha_{2}$	145	145	154	154	160	154
30 months of exposure in Gelendzhik	$\alpha_{1}$	117	117	119	121	124	128
	$\alpha_{2}$	148	150	152	155	162	160
28 months of exposure in Yakutsk	$\alpha_{1}$	115	116	119	121	125	126
	$\alpha_{2}$	155	160	159	163	165	164

The transition of the epoxy matrix from the glassy state to the highly elastic one occurs in a temperature range 90–190 °C both in the initial state and after the climatic tests in Gelendzhik and Yakutsk. During this time of action, rigidity of the armature remains high, and the polymer matrix is in the glassy state.

**Fig. 8:**
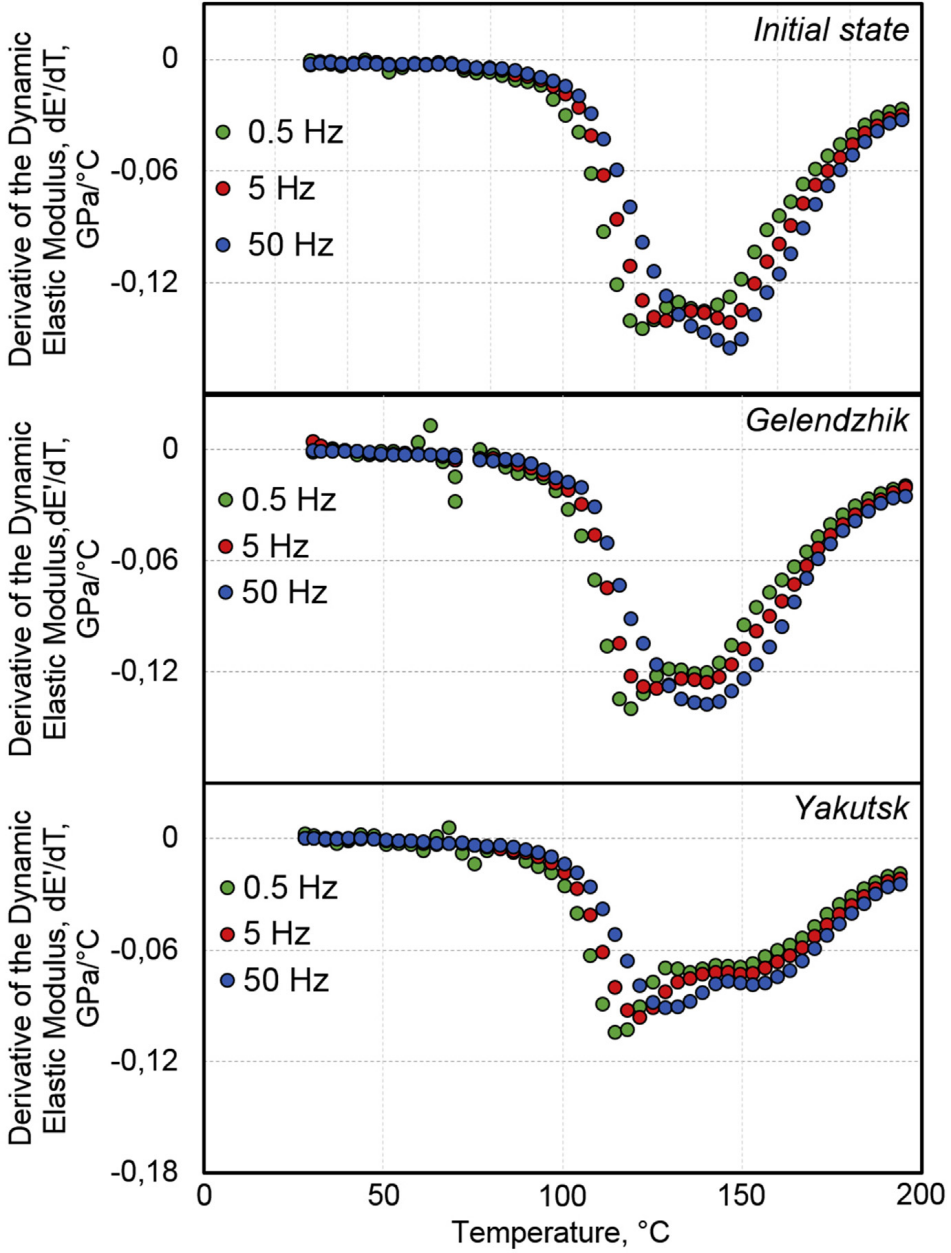
Effect of frequency of DMA measurements on temperature dependence of derivative of dynamic elastic modulus upon transition of epoxy binder of BPA from glassy state to highly elastic one: initial state, after 30 months of exposure in Gelendzhik, and after 28 months of exposure in Yakutsk. DMA measurements are performed at frequencies of 0.5 Hz, 5 Hz, and 50 Hz.

**Fig. 9:**
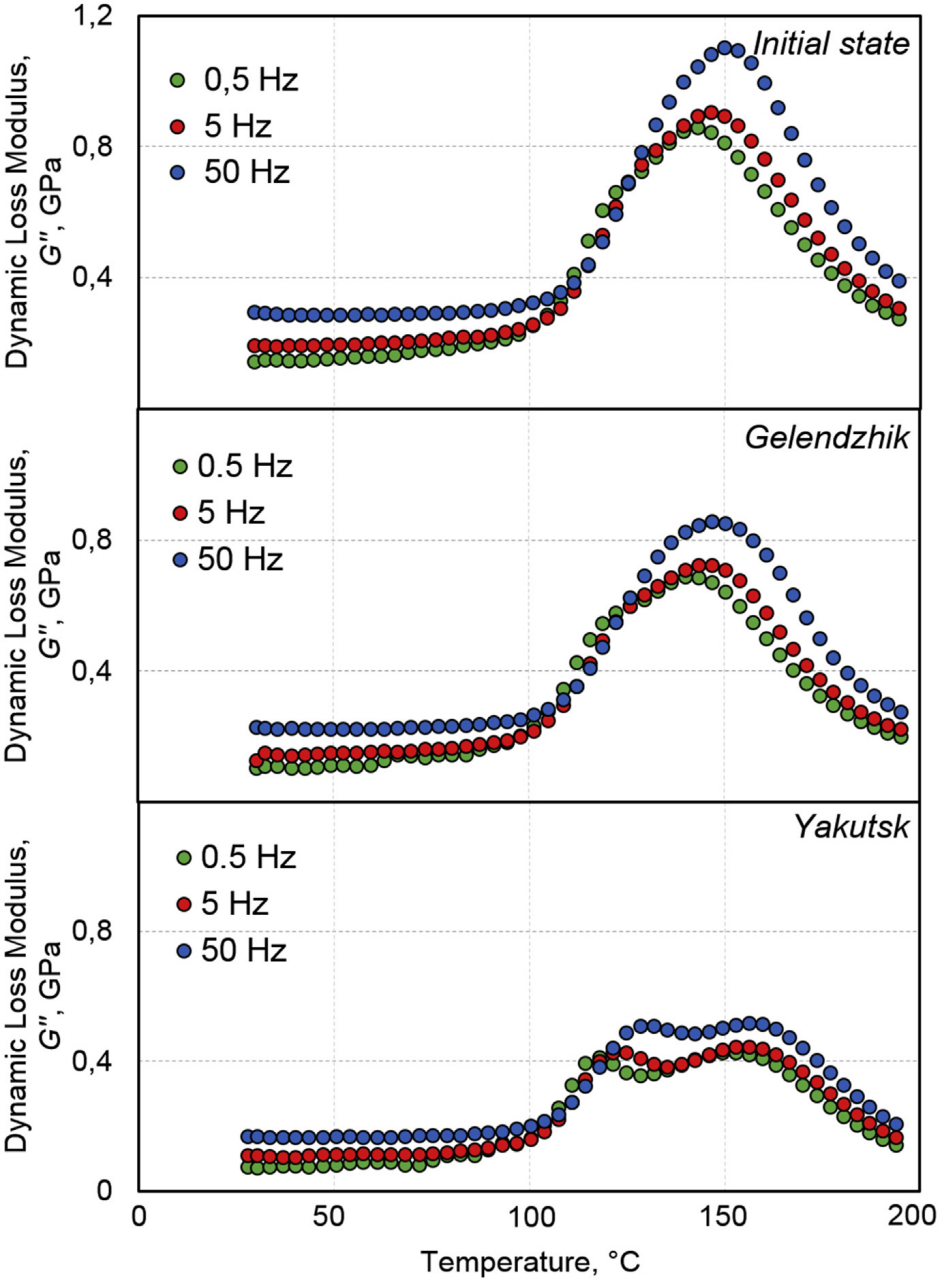
Effect of frequency of DMA measurements on temperature dependence of dynamic loss modulus upon transition of epoxy binder of BPA from glassy state to highly elastic one: initial state, after 30 months of exposure in Gelendzhik, and after 28 months of exposure in Yakutsk. DMA measurements are performed at frequencies of 0.5 Hz, 5 Hz, and 50 Hz.

Analysis of the dynamic mechanical characteristics of BPA reveals asymmetry of the transition of the epoxy matrix from the glassy state to the highly elastic one (the α-transition). Regularities of a change of mobility of kinetic elements under the influence of environmental factors are reproduced when the oscillation frequency changes by two orders of magnitude: from 0.5 Hz to 50 Hz (Figs. 8 and 9).

In the initial state, a double minimum of $dE^{\prime }/dT$ was revealed: the $\alpha_{1}$-minimum at 118 °C and the $\alpha_{2}$-minimum at 149 °C (Fig. 7). Such a double transition is characteristic of epoxy polymers. The detected transitions have a well-pronounced relaxation character: with an increase of the frequency from 0.5 Hz to 50 Hz, the temperatures of $\alpha_{1}$-minimum and $\alpha_{2}$-minimum shift, respectively, to 129 °C and 154 °C. The temperatures of these transitions are reproduced to within 2 °C in terms of the maxima of $E^{\prime \prime }$ in Fig. 9. After the exposure of BPA in the open climatic conditions, multiplicity of the transition was more pronounced (Figs. 7, 8, and 9). A tendency was found to shift the $\alpha_{1}$-transition to low temperatures, and the $\alpha_{2}$-transition to higher temperatures. Quantitative effects of these shifts are presented in Table 7.

According to data of Table 7, the effect of the decrease of the temperature of the $\alpha_{1}$-transition does not exceed 3 °C after the exposure in Gelendzhik and Yakutsk. A probable cause of this effect is weakening of intermolecular interaction in the surface layer of the composite. A check of action of the plasticizing effect of moisture shows that the initial and exposed samples had practically the same insignificant amount of moisture (0.1%–0.2%). Therefore, a decrease of the glass transition temperature is not associated with the effect of this factor.

As a result of the climatic action, the temperature of the $\alpha_{2}$–transition increased by 3–5 °C after 30 months of exposure in Gelendzhik and by 10–15 °C after the exposure in Yakutsk (Figs. 8 and 9, Table 7).

A reason for this increase of the temperature of the $\alpha_{2}$–transition is the post-curing of the polymer matrix, which occurs more actively in the climatic conditions of Yakutsk.

Distributions of the duration of sample exposure at certain temperatures have been obtained by direct measurements of the temperature in the samples obtained. The results show that in the summer months more than 120 hours of overheating of the sample temperature above 50 °C have been fixed in Yakutsk, while in Gelendzhik overheating has been fixed as about 50 hours.

It can be expected that structuring of the polymer matrix is a cause of unequal changes of the strength characteristics of BPA after the exposure in two different climatic zones. The post-curing of the epoxy matrix increases adhesive strength of fiber-matrix compounds during the exposure and contributes to enhancement of the strength properties of BPA. The matrix makes the material monolithic, promotes an efficient use of the mechanical properties of fibers and a uniform distribution of forces between them. The binder protects the fibers from chemical, atmospheric and other external influences, and also takes itself part of forces developing in the material at work under the load. This is also evidenced by a kind of failures of the samples. When testing the initial BPA samples, the failure occurred according to a “panicle” type, and after the exposure according to a “cut” type, which indirectly confirms the increase of the adhesive strength of the “fiber-matrix” compound.

### 3.3 Thermal expansion data

**Fig. 10:**
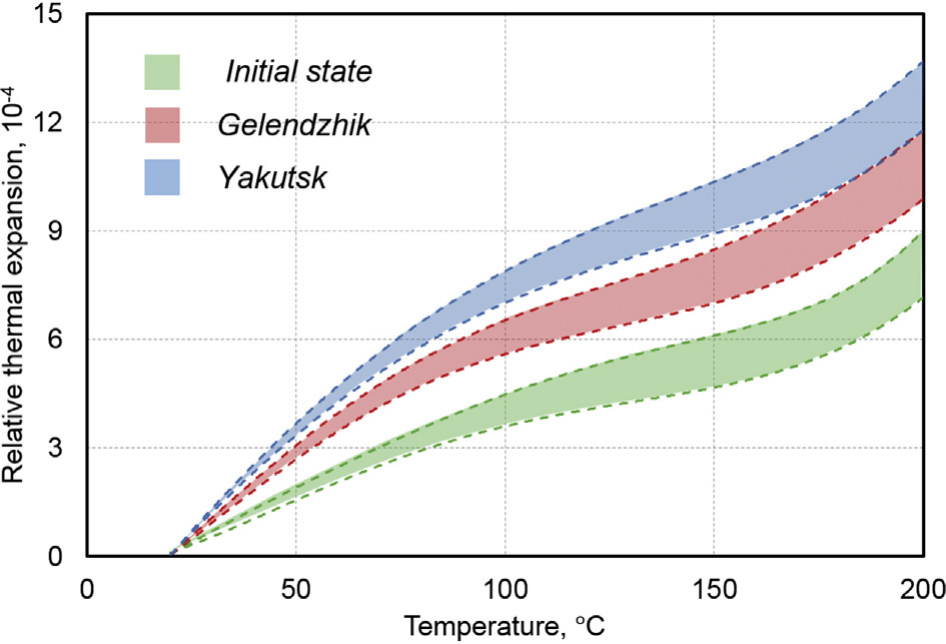
Temperature dependences of relative thermal expansion along direction of reinforcement of BPA in initial state, after 30 months of exposure in Gelendzhik, and 28 months of exposure in Yakutsk. Bands show spreads in measurements of three parallel samples.

The temperature dependences of a relative thermal expansion $\Delta L/L_{0}$ along a direction of reinforcement of BPA samples with a diameter of 20 mm and a height of 10 mm in the initial state and after the exposure in Gelendzhik and Yakutsk are shown in Fig. 10. To evaluate reproducibility of results, the measurements were performed on three parallel samples in each state. Measured values of $\Delta L/L_{0}$ in Fig. 10 are marked with colored stripes.

The temperature dependences of $\Delta L/L_{0}$ show the fairly good reproducibility. With an increase of the temperature from the room temperature to 120 ± 5 °C, the initial and exposed BPA samples show an increase of the relative thermal expansion. Such a relationship is characteristic of PCM, in which the polymer matrix is in the glassy state [20]. A character of this dependence changes at the temperature of 120 ± 5 °C. According to the data obtained by the DMA method (Figs. 7, 8, and 9, Table 7), it is here, where the glass transition temperature ($\alpha_{1}$-transition) of the epoxy matrix is located. After a slight shrinkage in the range 120–150 °C, further thermal expansion occurs, but with an increase of the coefficient of linear thermal expansion (CLTE) $\beta =\left(\frac{\Delta L}{L_{0}}\right)/\Delta T$.

The measurement results shown in Fig. 10 allow not only justifying the high stability of BPA in terms of parameters of thermal expansion, but also assessing subtle effects of the measured values for the parallel samples in the initial state and after the climatic tests in Gelendzhik and Yakutsk. The results of measurements of the thermal expansion of BPA are shown in Table 8. A calculation of CLTE β was performed under an assumption of a linear increase of $\Delta L/L_{0}$ at $T<T_{g}$ and $T>T_{g}$.

**Table 8:** Effect of exposure on parameters of linear expansion of BPA.

Conditions of climate tests	Relative thermal expansion in temperature interval 25–200°С, $\left(\Delta L/L_{0}\right)\cdot 10^{-4}$	Linear thermal expansion coefficient $\beta \cdot 10^{-6},\;K^{-1}$	
		$T<T_{g}$	$T>T_{g}$
Initial state	7–9.3	5.0–6.3	5.6–5.9
30 months of exposure in Gelendzhik	9.1–13	7.1–7.4	9.8–12.5
28 months of exposure in Yakutsk	12–14	7.7–9.3	7.5–9.5

A reason for the observed increase of $\Delta L/L_{0}$ and β is predominance of an effect of weakening of internal stresses of the polymer matrix at the interface with the basalt fiber over an effect of the post-curing of the polymer matrix of BPA. Similar patterns were previously observed in the climatic aging of glass-reinforced plastic VPS-7 on the basis of epoxy binder EDT-10P [20].

### 3.4 Sorption and diffusion of water

In BPA with the diameters of 6–20 mm, a variation of the sizes of the samples from 2 to 100 mm made it possible to reveal the effects of the influence of the periodic screw profile and the damaged surface layer formed during rod cutting on the maximum moisture content $M_{\infty }$ and the moisture diffusion coefficient *D*.

**Fig. 11:**
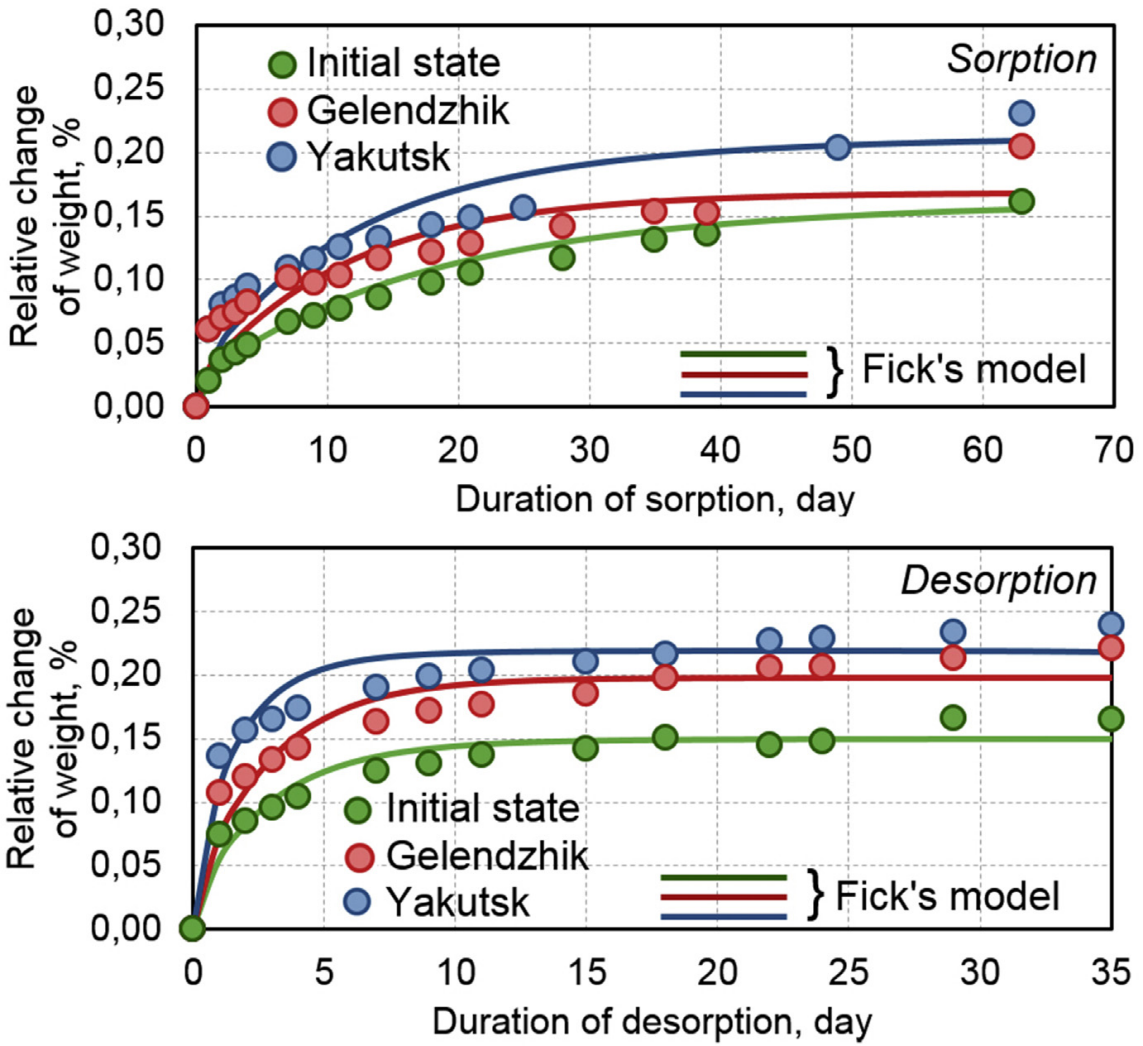
Kinetics of sorption and desorption of moisture in samples of BPA with diameter of 20 mm and length of 100 mm in initial state, after 30 months of exposure in Gelendzhik, and after 28 months of exposure in Yakutsk. Lines are approximation according to Fick's model of sorption (3–4).

Fig. 11 shows examples of sorption and subsequent desorption of moisture into the samples of the rods with the diameter of 20 mm and the length of 100 mm in the initial state and after the exposure in Gelendzhik and Yakutsk. For these samples and the samples of other sizes, a general pattern is observed: after 30 months of exposure in Gelendzhik, *w* increases. This parameter increases even more noticeably after 28 months of the exposure in Yakutsk. Analysis showed that kinetic curves of a change of the mass of the samples during the sorption and desorption of moisture are satisfactorily approximated by the second Fick law in a one-dimensional approximation with constant boundary conditions similar to [21]:

(3) $$\begin{array}{l} \frac{\partial \text{с}}{\partial t}=D\left(\frac{\partial^{2}\text{с}}{\partial r^{2}}+\frac{1}{r}\frac{\partial \text{с}}{\partial r}+\frac{\partial^{2}\text{с}}{\partial z^{2}}\right)\\ {\text{с}\left(r,\;t,z\right)|}_{t=0}=0\;\left(0<r<R,\;0<z<h\right)\\ {\text{с}\left(r,\;t,z\right)|}_{\begin{array}{c} r=R\\ z=h \end{array}}={\text{с}}_{0} \end{array}$$

where c is a concentration of moisture per unit volume of sample; $c_{0}$ is an initial moisture concentration at t→0; t is the time; *R* is a radius of the sample, *h* is a sample length, D is the coefficient diffusion. Parameters of Fick's desorption in the one-dimensional approximation (a maximum mass change, the diffusion coefficient) for cylindrical samples are found using a relationship [21]:

(4) $$M\left(t\right)=M_{\infty }\left(1-8\overset{\infty }{\underset{k=0}{\sum }}\frac{1}{n_{k}^{2}}e^{-n_{k}^{2}dt}\right)$$

where M(t) is a moisture content of a model segment of a length *λ* at a moment of time *t* (%), $M_{\infty }$ is the maximum moisture content of the samples, $d=2\pi R\left(R+h\right)\left(D/\lambda^{2}\right)$, $\lambda^{2}=\;\frac{R^{2}h^{2}}{R^{2}+h^{2}}$, *λ* is a characteristic length of a diffusion path, $n_{k}=\left(2k+1\right).$

In Table 9 for comparison, numerical values of the moisture diffusion coefficient and the maximum moisture content determined for the sorption and desorption of moisture according to formulas (4) for the samples of BPA, cut from the rods with the diameters of 6 and 20 mm.

**Table 9:** Effect of diameter and sizes of samples of BPA on moisture diffusion coefficient D·10^−4^ cm^2^/day (numerator) and maximum moisture saturation *M*_∞_, % (denominator) in initial state, after 30 months of exposure in Gelendzhik, and after 28 months of exposure in Yakutsk.

Diameter of sample, mm	Type of test	Place of climate exposure	Length of sample, mm						
			2	5	10	30	50	70	100
6	Sorption	Initial	$\frac{34}{0.44}$	$\frac{25}{0.41}$	$\frac{15}{0.38}$	$\frac{9.8}{0.4}$	$\frac{7.0}{0.28}$	$\frac{2.3}{0.23}$	$\frac{1.4}{0.21}$
		Gelendzhik	$\frac{35}{0.58}$	$\frac{24}{0.48}$	$\frac{19}{0.37}$	$\frac{17}{0.20}$	$\frac{12}{0.20}$	$\frac{5.2}{0.19}$	$\frac{3.1}{0.17}$
		Yakutsk	$\frac{41}{0.52}$	$\frac{27}{0.43}$	$\frac{21}{0.34}$	$\frac{16}{0.32}$	$\frac{11}{0.27}$	$\frac{4.5}{0.25}$	$\frac{3.7}{0.21}$
	Desorption	Initial	$\frac{34}{0.58}$	$\frac{33}{0.46}$	$\frac{25}{0.40}$	$\frac{18}{0.31}$	$\frac{13}{0.31}$	$\frac{6.4}{0.25}$	$\frac{5.7}{0.25}$
		Gelendzhik	$\frac{45}{0.68}$	$\frac{34}{0.58}$	$\frac{31}{0.50}$	$\frac{18}{0.40}$	$\frac{13}{0.40}$	$\frac{9.7}{0.10}$	$\frac{8.9}{0.30}$
		Yakutsk	$\frac{51}{0.68}$	$\frac{37}{0.61}$	$\frac{29}{0.54}$	$\frac{21}{0.48}$	$\frac{15}{0.43}$	$\frac{10}{0.36}$	$\frac{9.7}{0.34}$
20	Sorption	Initial	$\frac{17}{0.33}$	$\frac{13}{0.27}$	$\frac{8.5}{0.29}$	$\frac{2.5}{0.30}$	$\frac{2.5}{0.30}$	$\frac{2.1}{0.18}$	$\frac{0.88}{0.16}$
		Gelendzhik	$\frac{33}{0.31}$	$\frac{22}{0.29}$	$\frac{14}{0.30}$	$\frac{2.9}{0.27}$	$\frac{2.9}{0.27}$	$\frac{2.2}{0.19}$	$\frac{1.2}{0.18}$
		Yakutsk	$\frac{21}{0.32}$	$\frac{17}{0.34}$	$\frac{14}{0.33}$	$\frac{2.6}{0.30}$	$\frac{2.6}{0.30}$	$\frac{2.3}{0.23}$	$\frac{1.5}{0.22}$
	Desorption	Initial	$\frac{21}{0.34}$	$\frac{15}{0.33}$	$\frac{11}{0.33}$	$\frac{7.6}{0.30}$	$\frac{7.0}{0.22}$	$\frac{5.7}{0.19}$	$\frac{4.5}{0.16}$
		Gelendzhik	$\frac{37}{0.37}$	$\frac{21}{0.35}$	$\frac{17}{0.35}$	$\frac{9.9}{0.34}$	$\frac{8.8}{0.30}$	$\frac{7.5}{0.24}$	$\frac{5.6}{0.22}$
		Yakutsk	$\frac{32}{0.38}$	$\frac{28}{0.37}$	$\frac{22}{0.37}$	$\frac{11}{0.35}$	$\frac{9.7}{0.24}$	$\frac{8.6}{0.22}$	$\frac{7.1}{0.24}$

After the exposure in the full-scale climatic conditions, the moisture transfer parameters increase. A general regularity is an increase of the values of $M_{\infty }$ and D at a drying stage of the moisture-saturated samples in comparison with the corresponding values of these parameters under humidification due to physicochemical transformations in the epoxy matrix of BPA with moisture plasticisation [19]. Similar effects were observed earlier for other PCMs based on the epoxy polymers [21]. A significant increase of the values of $M_{\infty }$ and D with a decrease of the length of the samples is caused by influence of a defective end edge formed when cutting the samples [21]. In pores and defects of the edge, more moisture is sorbed than in undamaged parts of the sample. Therefore, the shorter the sample, the greater the role of edge effects, and the parameters $M_{\infty }$ and D increase in these samples. A more detailed analysis of influence of the edge effects on the characteristics of moisture transfer in BPA after the exposure in the climatic conditions will be presented in another report.

Results presented in Table 9 show that $M_{\infty }$ and D increase with a decrease of the diameter of the samples. The surface relief screw layer of BPA sorbs more moisture than inner tightly packed (by the basalt fibers) layers of the rods. With the decrease of the diameter of the rod, part of the surface relief layer in the volume of the samples increases, which leads to the increase of $M_{\infty }$ and D.

The parameters $M_{\infty }$ and D increase to a greater extent after the exposure in Yakutsk than in Gelendzhik. The exposure in the climatic conditions has an unequal effect on the characteristics of the moisture transfer of BPA of different sections. For example, for the samples of the length of 100 mm and the diameter of 20 mm after 30 months of the exposure in Gelendzhik, the moisture diffusion coefficient with the sorption increases by 36% after 30 months of the exposure in Gelendzhik and by 71% after 28 months of the exposure in Yakutsk. For the rods of the same length and with the diameter of 6 mm, a similar increase is 120% and 160%, respectively (Table 9). Thus, a general conclusion can be drawn that the characteristics of moisture transfer are the parameters sensitive to physicochemical aging processes in the surface layer of BPA.

### 3.5 Optical microscopy, fractography

**Fig. 12:**
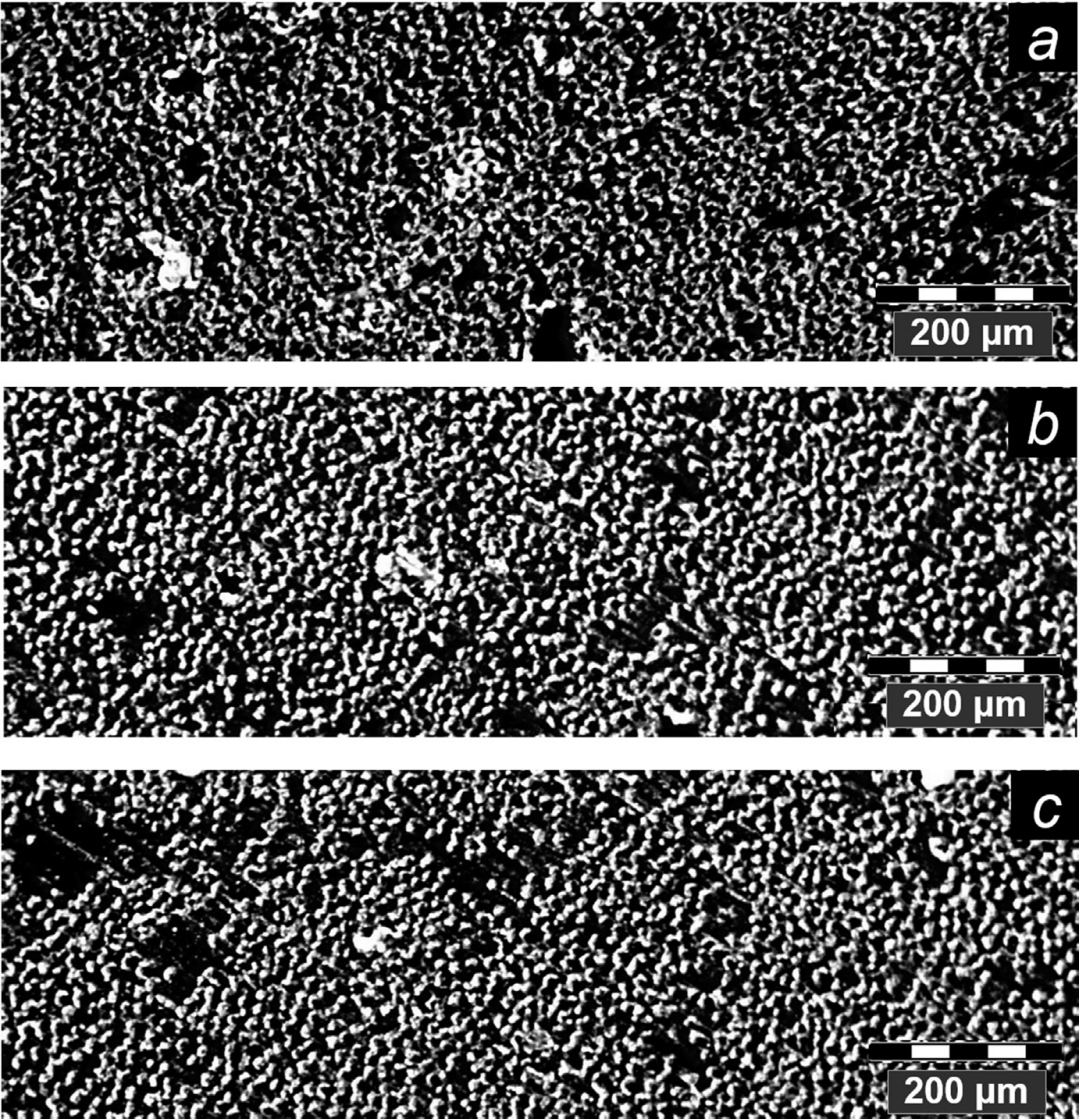
Fragments of polished sections of BPA with cross section of 20 mm in initial state (a), after 30 months of exposure in Gelendzhik (b), and after 28 months of exposure in Yakutsk (c).

According to data of fractographic analysis, the structure of BPA after the exposure in the climatic conditions does not undergo significant changes. As an example, Fig. 12 shows fragments of microsections of BPA with the diameter of 20 mm in the initial state, after 30 months of the exposure in Gelendzhik and after 28 months of the exposure in Yakutsk. In the initial state, the structure of BPA is quite monolithic. A dense chaotic structure of reinforcing basalt fibers is traced. Rare pores with a size of up to 10–20 μm are noted. After the exposure in the climatic conditions, the amount and size of pores increases by 20–40%. A slight increase of porosity is caused by thermal deformations of BPA with daily and seasonal changes of temperature. This increase of porosity explains the increase of the maximum moisture saturation and the coefficient of moisture diffusion after the climatic action.

## 4 Conclusions

In this article the experimental study of the properties of rods of BPA of periodic profile with the diameters of 6, 8, 10, 16, and 20 mm on the basis of the epoxy matrix in the initial state and after the exposure during 30 months on the open stands in the moderately warm marine climate of Gelendzhik and 28 months on similar stands in the extremely cold climate of Yakutsk. From the analysis of the results obtained, the following conclusions can be drawn:

1. After the exposure in Yakutsk, the increase of the ultimate compression strength by 4–12% was found, depending on the diameter of the rods. After the exposure in Gelendzhik this parameter decreases by 10–17%.
2. The method of dynamic mechanical analysis revealed the transition from the glassy state to the highly elastic one of the epoxy matrix of BPA: the $\alpha_{1}$-transition at 118 °C and the $\alpha_{2}$-transition at 149 °C. After the climatic action, the shifts of the $\alpha_{1}$-transition to low temperatures and the $\alpha_{2}$-transition to higher temperatures were detected. The reasons for the change of the temperatures of the transitions are the weakening of the intermolecular interaction and the post-curing of the epoxy matrix in the surface layer.
3. A method of linear dilatometry showed the increase in the coefficient of linear thermal expansion of the rods along the reinforcement direction after the climatic action. Due to the action of cyclic temperatures, humidity, solar radiation during the climatic exposure, the effect of weakening of internal stresses of the polymer matrix at the interface with the basalt fiber predominates over the post-curing effect.
4. After the exposure in the full-scale climatic conditions, the maximum moisture saturation $M_{\infty }$ and the moisture diffusion coefficient D increase in BPA due to the physicochemical transformations during the moisture plasticization of the epoxy matrix. These parameters increase to the greater extent after the exposure in Yakutsk than in Gelendzhik.
5. The exposure in the climatic conditions has an uneven effect on the characteristics of the moisture transfer of BPA of different cross-sections. The surface relief screw layer of BPA sorbs more moisture than the inner tightly packed (by the basalt fibers) layers of the rods. With the decrease of the diameter of the rod, the part of the surface relief layer in the volume of the samples increases, which leads to the increase of $M_{\infty }$ and D.
6. The fractographic studies discovered the presence of pores with the sizes up to 10–20 μm in the structure of BPA. The quantity and size of pores increase by 20–40% after the climatic exposure.
7. The studied BPA has the high climatic stability and can be used for the long time under the extreme climatic conditions.

## Declarations

### Author contribution statement

Valery Startsev: Conceived and designed the experiments; Performed the experiments; Analyzed and interpreted the data; Contributed reagents, materials, analysis tools or data; Wrote the paper.

Mikhail Lebedev: Analyzed and interpreted the data; Wrote the paper.

Anatoly Kychkin: Performed the experiments; Contributed reagents, materials, analysis tools or data.

### Funding statement

This work was supported by the framework of implementation of a project of complex scientific studies in the Republic of Sakha (Yakutia), aimed at development of productive forces and social sphere for 2016–2020 in direction 4 «Ensuring economic development» on a theme «Complex climate tests of materials under conditions of atmosphere of Arctic climate and in marine environment».

### Competing interest statement

The authors declare no conflict of interest.

### Additional information

No additional information is available for this paper.
